# *N*-(2-Chloro­phen­yl)-2,4-dimethyl­benzene­sulfonamide

**DOI:** 10.1107/S1600536810015916

**Published:** 2010-05-08

**Authors:** B. Thimme Gowda, Sabine Foro, P. G. Nirmala, Hartmut Fuess

**Affiliations:** a Department of Chemistry, Mangalore University, Mangalagangotri 574 199, Mangalore, India; b Institute of Materials Science, Darmstadt University of Technology, Petersenstrasse 23, D-64287 Darmstadt, Germany

## Abstract

In the title compound, C_14_H_14_ClNO_2_S, the conformation of the N—C bond in the C—SO_2_—NH—C segment has *gauche* torsions with respect to the S=O bonds. The mol­ecule is bent at the S atom with a C—SO_2_—NH—C torsion angle of −54.9 (2)°. The sulfonyl and aniline benzene rings are rotated relative to each other by 75.7 (1)°. An intra­molecular N—H⋯Cl hydrogen bond is present. In the crystal, inter­molecular N—H⋯O hydrogen-bonding inter­actions are observed and the mol­ecules are packed into chains parallel to the *b* axis.

## Related literature

For the preparation of the title compound, see: Savitha & Gowda (2006 ▶). For related structures, see: Gelbrich *et al.* (2007 ▶); Gowda *et al.* (2009**a* ▶,b* ▶, 2010 ▶); Nirmala *et al.* (2009 ▶); Perlovich *et al.* (2006 ▶).
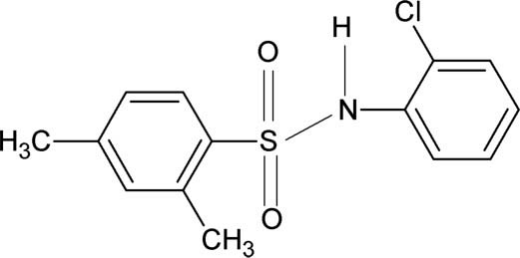


## Experimental

### 

#### Crystal data

C_14_H_14_ClNO_2_S
*M* *_r_* = 295.77
Orthorhombic, 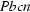

*a* = 10.574 (1) Å
*b* = 16.269 (2) Å
*c* = 16.859 (2) Å
*V* = 2900.2 (6) Å^3^
*Z* = 8
Mo *K*α radiation
μ = 0.40 mm^−1^
*T* = 299 K
0.38 × 0.34 × 0.24 mm

#### Data collection

Oxford Diffraction Xcalibur diffractometer with a Sapphire CCD detector
Absorption correction: multi-scan (*CrysAlis RED*; Oxford Diffraction, 2009 ▶) *T*_min_ = 0.862, *T*_max_ = 0.909
18999 measured reflections
2922 independent reflections
2330 reflections with *I* > 2σ(*I*)
*R*_int_ = 0.026

#### Refinement

*R*[*F* ^2^ > 2σ(*F* ^2^)] = 0.037
*wR*(*F* ^2^) = 0.102
*S* = 1.05
2922 reflections
178 parameters
1 restraint
H atoms treated by a mixture of independent and constrained refinement
Δρ_max_ = 0.23 e Å^−3^
Δρ_min_ = −0.27 e Å^−3^

### 

Data collection: *CrysAlis CCD* (Oxford Diffraction, 2009 ▶); cell refinement: *CrysAlis RED* (Oxford Diffraction, 2009 ▶); data reduction: *CrysAlis RED*; program(s) used to solve structure: *SHELXS97* (Sheldrick, 2008 ▶); program(s) used to refine structure: *SHELXL97* (Sheldrick, 2008 ▶); molecular graphics: *PLATON* (Spek, 2009 ▶); software used to prepare material for publication: *SHELXL97*.

## Supplementary Material

Crystal structure: contains datablocks I, global. DOI: 10.1107/S1600536810015916/rz2441sup1.cif

Structure factors: contains datablocks I. DOI: 10.1107/S1600536810015916/rz2441Isup2.hkl

Additional supplementary materials:  crystallographic information; 3D view; checkCIF report

## Figures and Tables

**Table 1:** Hydrogen-bond geometry (Å, °)

*D*—H⋯*A*	*D*—H	H⋯*A*	*D*⋯*A*	*D*—H⋯*A*
N1—H1*N*⋯O1^i^	0.85 (1)	2.20 (1)	3.010 (2)	159 (2)
N1—H1*N*⋯Cl1	0.85 (1)	2.63 (2)	2.9822 (18)	106 (2)

Symmetry code: (i) 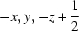.

**Fig. 1.**
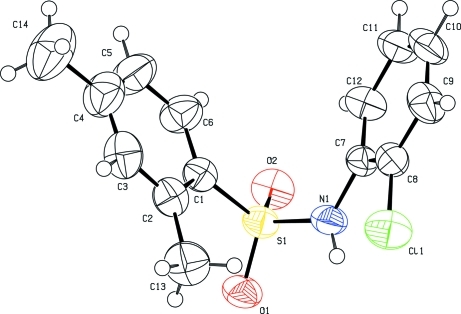
Molecular structure of (I), showing the atom labelling scheme and displacement ellipsoids drawn at the 50% probability level.

**Fig. 2.**
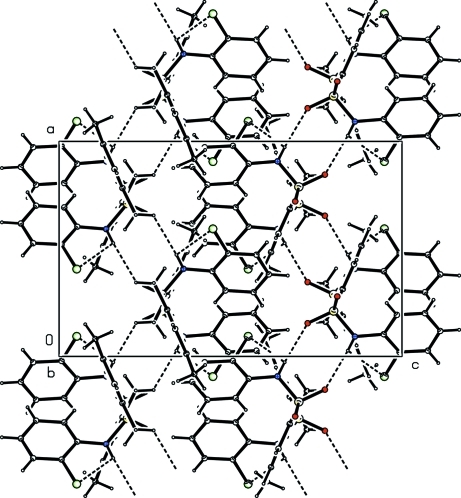
Molecular packing of (I) with hydrogen bonding shown as dashed lines.

## supplementary crystallographic information

### Comment

As part of a study of the substituent effects on the structures of *N*-(aryl)-arylsulfonamides (Gowda *et al. *, 2009*a*,*b*, 2010), in the present work the structure of 2,4-dimethyl-*N*-(2-chlorophenyl)benzenesulfonamide (I) has been determined (Fig. 1). The conformation of the N—C bond in the C—SO_2_—NH—C segment has *gauche* torsions with respect to the S═O bonds. The molecule is bent at the S atom with the C1—SO_2_—NH—C7 torsion angle of -54.9 (2)°, compared to the values of 71.6 (1)° in 2,4-dimethyl-*N*-(2-methylphenyl)benzenesulfonamide (II) (Nirmala *et al.*, 2009), 74.8 (4)° in 4-chloro-2-methyl-*N*-(2-chlorophenyl)benzenesulfonamide (III) (Gowda *et al.*, 2009*b*), -46.1 (3)° (molecule 1) and 47.7 (3)° (molecule 2) in the two molecules of 2,4-dimethyl-*N*-(phenyl)benzenesulfonamide (IV) (Gowda *et al.*, 2009*a*) and -54.8 (2)° in *N*-(2-chlorophenyl)-4-methylbenzenesulfonamide (V) (Gowda *et al.*, 2010).

The two benzene rings in (I) are tilted relative to each other by 75.7 (1)°, compared to the values of 47.0 (1)° (II), 45.5 (2)° in (III), 67.5 (1)° in molecule 1 and 72.9 (1)° in molecule 2 of (IV), and 71.6 (1)° in (V). The other bond parameters in (I) are similar to those observed in (II), (III), (IV), (V) and other aryl sulfonamides (Perlovich *et al.*, 2006; Gelbrich *et al.*, 2007).

The structure shows simultaneous N—H···Cl intramolecular and N—H···O intermolecular H-bonding (Table 1). The crystal packing of molecules in (I) *via* N—H···O(S) hydrogen bonds is shown in Fig.2.

### Experimental

A solution of 1,3-xylene (1,3-dimethylbenzene) (10 ml) in chloroform (40 ml) was treated dropwise with chlorosulfonic acid (25 ml) at 0 °C. After the initial evolution of hydrogen chloride subsided, the reaction mixture was brought to room temperature and poured into crushed ice in a beaker. The chloroform layer was separated, washed with cold water and allowed to evaporate slowly. The residual 2,4-dimethylbenzenesulfonylchloride was treated with 2-chloroaniline in the stoichiometric ratio and boiled for ten minutes. The reaction mixture was then cooled to room temperature and added to ice cold water (100 cc). The resultant solid 2,4-dimethyl-*N*-(2-chlorophenyl)benzenesulfonamide was filtered under suction and washed thoroughly with cold water. It was then recrystallized to constant melting point from dilute ethanol. The purity of the compound was checked and characterized by recording its infrared and NMR spectra (Savitha & Gowda, 2006). The prism like colourless single crystals used in X-ray diffraction studies were grown by slow evaporation of an ethanol solution at room temperature.

### Refinement

The H atom of the NH group was located in a difference map and later restrained to N—H = 0.86 (1) %A. The other H atoms were positioned with idealized geometry using a riding model with C—H = 0.93–0.96 Å. All H atoms were refined with isotropic displacement parameters (set to 1.2 times of the *U*_eq_ of the parent atom).

### Crystal data

**Table:**

C_14_H_14_ClNO_2_S	*F*(000) = 1232
*M**_r_* = 295.77	*D*_x_ = 1.355 Mg m^−^^3^
Orthorhombic, *P**b**c**n*	Mo *K*α radiation, λ = 0.71073 Å
Hall symbol: -P 2n 2ab	Cell parameters from 2348 reflections
*a* = 10.574 (1) Å	θ = 2.6–27.8°
*b* = 16.269 (2) Å	µ = 0.40 mm^−^^1^
*c* = 16.859 (2) Å	*T* = 299 K
*V* = 2900.2 (6) Å^3^	Prism, colourless
*Z* = 8	0.38 × 0.34 × 0.24 mm

### Data collection

**Table:**

Oxford Diffraction Xcalibur diffractometer with a Sapphire CCD detector	2922 independent reflections
Radiation source: fine-focus sealed tube	2330 reflections with *I* > 2σ(*I*)
graphite	*R*_int_ = 0.026
Rotation method data acquisition using ω and phi scans	θ_max_ = 26.4°, θ_min_ = 2.6°
Absorption correction: multi-scan (*CrysAlis RED*; Oxford Diffraction, 2009)	*h* = −8→13
*T*_min_ = 0.862, *T*_max_ = 0.909	*k* = −19→19
18999 measured reflections	*l* = −20→20

### Refinement

**Table:**

Refinement on *F*^2^	Secondary atom site location: difference Fourier map
Least-squares matrix: full	Hydrogen site location: inferred from neighbouring sites
*R*[*F*^2^ > 2σ(*F*^2^)] = 0.037	H atoms treated by a mixture of independent and constrained refinement
*wR*(*F*^2^) = 0.102	*w* = 1/[σ^2^(*F*_o_^2^) + (0.0452*P*)^2^ + 1.1956*P*] where *P* = (*F*_o_^2^ + 2*F*_c_^2^)/3
*S* = 1.05	(Δ/σ)_max_ = 0.003
2922 reflections	Δρ_max_ = 0.23 e Å^−^^3^
178 parameters	Δρ_min_ = −0.27 e Å^−^^3^
1 restraint	Extinction correction: *SHELXL97* (Sheldrick, 2008), Fc^*^=kFc[1+0.001xFc^2^λ^3^/sin(2θ)]^-1/4^
Primary atom site location: structure-invariant direct methods	Extinction coefficient: 0.0063 (6)

### Special details

**Table:**

Experimental. CrysAlis RED (Oxford Diffraction, 2009) Empirical absorption correction using spherical harmonics, implemented in SCALE3 ABSPACK scaling algorithm.
Geometry. All esds (except the esd in the dihedral angle between two l.s. planes) are estimated using the full covariance matrix. The cell esds are taken into account individually in the estimation of esds in distances, angles and torsion angles; correlations between esds in cell parameters are only used when they are defined by crystal symmetry. An approximate (isotropic) treatment of cell esds is used for estimating esds involving l.s. planes.
Refinement. Refinement of *F*^2^ against ALL reflections. The weighted *R*-factor wR and goodness of fit *S* are based on *F*^2^, conventional *R*-factors *R* are based on *F*, with *F* set to zero for negative *F*^2^. The threshold expression of *F*^2^ > σ(*F*^2^) is used only for calculating *R*-factors(gt) etc. and is not relevant to the choice of reflections for refinement. *R*-factors based on *F*^2^ are statistically about twice as large as those based on *F*, and *R*- factors based on ALL data will be even larger.

### Fractional atomic coordinates and isotropic or equivalent isotropic displacement parameters (Å^2^)

**Table:**

	*x*	*y*	*z*	*U*_iso_*/*U*_eq_	
C1	0.31380 (18)	0.39372 (11)	0.32739 (11)	0.0470 (4)	
C2	0.2828 (2)	0.47702 (12)	0.31522 (12)	0.0543 (5)	
C3	0.3721 (2)	0.53467 (14)	0.33864 (15)	0.0707 (6)	
H3	0.3530	0.5900	0.3318	0.085*	
C4	0.4873 (3)	0.51455 (17)	0.37137 (16)	0.0809 (7)	
C5	0.5148 (3)	0.43261 (18)	0.38187 (19)	0.0872 (8)	
H5	0.5921	0.4175	0.4038	0.105*	
C6	0.4290 (2)	0.37243 (15)	0.36013 (15)	0.0670 (6)	
H6	0.4490	0.3173	0.3676	0.080*	
C7	0.10341 (18)	0.31392 (11)	0.44692 (10)	0.0465 (4)	
C8	0.0207 (2)	0.35953 (11)	0.49298 (11)	0.0499 (5)	
C9	0.0269 (3)	0.35663 (14)	0.57551 (12)	0.0660 (6)	
H9	−0.0305	0.3863	0.6059	0.079*	
C10	0.1184 (3)	0.30960 (14)	0.61177 (12)	0.0712 (7)	
H10	0.1237	0.3083	0.6668	0.085*	
C11	0.2011 (3)	0.26496 (15)	0.56722 (13)	0.0687 (6)	
H11	0.2633	0.2338	0.5921	0.082*	
C12	0.1932 (2)	0.26568 (14)	0.48522 (12)	0.0629 (6)	
H12	0.2485	0.2336	0.4555	0.076*	
C13	0.1609 (2)	0.50496 (15)	0.27754 (16)	0.0766 (7)	
H13A	0.0907	0.4846	0.3078	0.092*	
H13B	0.1560	0.4841	0.2244	0.092*	
H13C	0.1583	0.5639	0.2764	0.092*	
C14	0.5812 (3)	0.5810 (2)	0.3941 (3)	0.1248 (13)	
H14A	0.5396	0.6217	0.4260	0.150*	
H14B	0.6140	0.6063	0.3470	0.150*	
H14C	0.6494	0.5570	0.4237	0.150*	
N1	0.09250 (16)	0.31599 (11)	0.36289 (9)	0.0538 (4)	
H1N	0.0245 (14)	0.3331 (13)	0.3411 (12)	0.065*	
O1	0.15450 (14)	0.32896 (10)	0.22493 (7)	0.0610 (4)	
O2	0.27871 (14)	0.23745 (8)	0.31126 (9)	0.0625 (4)	
Cl1	−0.09351 (6)	0.42128 (4)	0.44867 (3)	0.0714 (2)	
S1	0.21116 (5)	0.31295 (3)	0.30076 (3)	0.04739 (16)	

### Atomic displacement parameters (Å^2^)

**Table:**

	*U*^11^	*U*^22^	*U*^33^	*U*^12^	*U*^13^	*U*^23^
C1	0.0497 (11)	0.0479 (10)	0.0435 (9)	0.0042 (8)	0.0033 (8)	−0.0029 (8)
C2	0.0586 (12)	0.0515 (11)	0.0528 (11)	0.0071 (9)	0.0096 (9)	−0.0013 (9)
C3	0.0839 (17)	0.0502 (12)	0.0779 (16)	−0.0034 (12)	0.0141 (14)	−0.0051 (11)
C4	0.0782 (17)	0.0775 (17)	0.0872 (18)	−0.0189 (14)	−0.0004 (15)	−0.0105 (14)
C5	0.0599 (14)	0.0923 (19)	0.109 (2)	−0.0086 (14)	−0.0208 (15)	0.0019 (16)
C6	0.0587 (13)	0.0597 (13)	0.0825 (16)	0.0057 (11)	−0.0130 (12)	0.0024 (11)
C7	0.0548 (11)	0.0488 (10)	0.0358 (9)	−0.0121 (9)	−0.0039 (8)	0.0009 (8)
C8	0.0643 (12)	0.0432 (9)	0.0423 (9)	−0.0129 (9)	−0.0035 (9)	−0.0022 (8)
C9	0.0950 (18)	0.0603 (13)	0.0428 (11)	−0.0148 (12)	0.0070 (11)	−0.0098 (9)
C10	0.112 (2)	0.0645 (14)	0.0372 (10)	−0.0271 (14)	−0.0138 (12)	0.0084 (10)
C11	0.0886 (17)	0.0681 (14)	0.0494 (12)	−0.0103 (13)	−0.0191 (12)	0.0122 (10)
C12	0.0715 (14)	0.0687 (14)	0.0487 (11)	0.0044 (11)	−0.0093 (10)	0.0056 (10)
C13	0.0767 (16)	0.0623 (14)	0.0907 (18)	0.0212 (13)	−0.0013 (14)	0.0076 (12)
C14	0.110 (3)	0.110 (3)	0.154 (3)	−0.048 (2)	−0.006 (2)	−0.023 (2)
N1	0.0493 (10)	0.0769 (12)	0.0352 (8)	0.0021 (9)	−0.0043 (7)	0.0025 (8)
O1	0.0647 (9)	0.0840 (10)	0.0342 (7)	0.0007 (8)	−0.0022 (6)	−0.0054 (6)
O2	0.0747 (10)	0.0478 (8)	0.0650 (9)	0.0094 (7)	−0.0006 (8)	−0.0098 (6)
Cl1	0.0827 (4)	0.0706 (4)	0.0610 (3)	0.0138 (3)	−0.0051 (3)	−0.0124 (3)
S1	0.0538 (3)	0.0511 (3)	0.0372 (2)	0.0036 (2)	−0.0007 (2)	−0.00583 (18)

### Geometric parameters (Å, °)

**Table:**

C1—C6	1.381 (3)	C9—C10	1.376 (3)
C1—C2	1.409 (3)	C9—H9	0.9300
C1—S1	1.7624 (19)	C10—C11	1.363 (3)
C2—C3	1.388 (3)	C10—H10	0.9300
C2—C13	1.508 (3)	C11—C12	1.385 (3)
C3—C4	1.377 (4)	C11—H11	0.9300
C3—H3	0.9300	C12—H12	0.9300
C4—C5	1.376 (4)	C13—H13A	0.9600
C4—C14	1.518 (4)	C13—H13B	0.9600
C5—C6	1.385 (3)	C13—H13C	0.9600
C5—H5	0.9300	C14—H14A	0.9600
C6—H6	0.9300	C14—H14B	0.9600
C7—C8	1.385 (3)	C14—H14C	0.9600
C7—C12	1.391 (3)	N1—S1	1.6352 (17)
C7—N1	1.422 (2)	N1—H1N	0.854 (9)
C8—C9	1.394 (3)	O1—S1	1.4357 (14)
C8—Cl1	1.739 (2)	O2—S1	1.4319 (14)
			
C6—C1—C2	120.29 (19)	C9—C10—H10	119.9
C6—C1—S1	117.24 (16)	C10—C11—C12	120.5 (2)
C2—C1—S1	122.47 (15)	C10—C11—H11	119.8
C3—C2—C1	116.8 (2)	C12—C11—H11	119.8
C3—C2—C13	119.8 (2)	C11—C12—C7	120.6 (2)
C1—C2—C13	123.37 (19)	C11—C12—H12	119.7
C4—C3—C2	123.7 (2)	C7—C12—H12	119.7
C4—C3—H3	118.1	C2—C13—H13A	109.5
C2—C3—H3	118.1	C2—C13—H13B	109.5
C5—C4—C3	118.0 (2)	H13A—C13—H13B	109.5
C5—C4—C14	121.3 (3)	C2—C13—H13C	109.5
C3—C4—C14	120.7 (3)	H13A—C13—H13C	109.5
C4—C5—C6	120.8 (2)	H13B—C13—H13C	109.5
C4—C5—H5	119.6	C4—C14—H14A	109.5
C6—C5—H5	119.6	C4—C14—H14B	109.5
C1—C6—C5	120.4 (2)	H14A—C14—H14B	109.5
C1—C6—H6	119.8	C4—C14—H14C	109.5
C5—C6—H6	119.8	H14A—C14—H14C	109.5
C8—C7—C12	118.24 (17)	H14B—C14—H14C	109.5
C8—C7—N1	119.64 (17)	C7—N1—S1	125.12 (14)
C12—C7—N1	122.10 (18)	C7—N1—H1N	120.3 (16)
C7—C8—C9	120.8 (2)	S1—N1—H1N	112.4 (15)
C7—C8—Cl1	120.47 (14)	O2—S1—O1	118.28 (9)
C9—C8—Cl1	118.77 (17)	O2—S1—N1	109.24 (9)
C10—C9—C8	119.7 (2)	O1—S1—N1	104.16 (9)
C10—C9—H9	120.1	O2—S1—C1	107.52 (9)
C8—C9—H9	120.1	O1—S1—C1	110.40 (9)
C11—C10—C9	120.2 (2)	N1—S1—C1	106.67 (9)
C11—C10—H10	119.9		
			
C6—C1—C2—C3	−0.9 (3)	Cl1—C8—C9—C10	−178.55 (16)
S1—C1—C2—C3	−179.84 (16)	C8—C9—C10—C11	−1.1 (3)
C6—C1—C2—C13	178.1 (2)	C9—C10—C11—C12	−0.7 (4)
S1—C1—C2—C13	−0.9 (3)	C10—C11—C12—C7	2.0 (4)
C1—C2—C3—C4	0.9 (3)	C8—C7—C12—C11	−1.4 (3)
C13—C2—C3—C4	−178.1 (2)	N1—C7—C12—C11	−179.9 (2)
C2—C3—C4—C5	−0.5 (4)	C8—C7—N1—S1	144.02 (16)
C2—C3—C4—C14	178.8 (3)	C12—C7—N1—S1	−37.6 (3)
C3—C4—C5—C6	0.0 (4)	C7—N1—S1—O2	61.07 (19)
C14—C4—C5—C6	−179.3 (3)	C7—N1—S1—O1	−171.66 (16)
C2—C1—C6—C5	0.5 (3)	C7—N1—S1—C1	−54.86 (19)
S1—C1—C6—C5	179.5 (2)	C6—C1—S1—O2	−6.68 (19)
C4—C5—C6—C1	0.0 (4)	C2—C1—S1—O2	172.29 (15)
C12—C7—C8—C9	−0.4 (3)	C6—C1—S1—O1	−137.03 (17)
N1—C7—C8—C9	178.06 (18)	C2—C1—S1—O1	41.94 (19)
C12—C7—C8—Cl1	179.82 (15)	C6—C1—S1—N1	110.40 (17)
N1—C7—C8—Cl1	−1.7 (2)	C2—C1—S1—N1	−70.63 (18)
C7—C8—C9—C10	1.7 (3)		

### Hydrogen-bond geometry (Å, °)

**Table:**

*D*—H···*A*	*D*—H	H···*A*	*D*···*A*	*D*—H···*A*
N1—H1N···O1^i^	0.85 (1)	2.20 (1)	3.010 (2)	159 (2)
N1—H1N···Cl1	0.85 (1)	2.63 (2)	2.9822 (18)	106 (2)

Symmetry codes: (i) −*x*, *y*, −*z*+1/2.
